# Beyond the Bench: Clean Sweep: Adopting Safer Urban Demolition Practices

**DOI:** 10.1289/ehp.115-a83a

**Published:** 2007-02

**Authors:** Tanya Tillett

In cities undergoing urban revitalization, progress can often be a costly benefit, particularly for residents living right in the midst of the changes. As staff at the Johns Hopkins NIEHS Center in Urban Environmental Health have documented, without proper standards in place, byproducts from urban renewal projects can be not only inconvenient, but also a threat to the health and safety of community members, particularly those living in low-income neighborhoods, where renewal projects tend to occur most often.

Since the 1970s, ongoing renewal efforts in Baltimore have improved the workplace, living, and leisure choices for city residents. But the measures used to revamp the city have also introduced environmental threats including dust, waste water, large amounts of uncontained debris, noise, vibration, and rats and other pests fleeing demolished buildings. In addition, residents have voiced concerns about the possible presence of lead in the dust and debris of demolished housing. So in 2000 the center began working with community members to assess the environmental health issues.

Under the direction of faculty member Mark Farfel, center staff collected vacuum sidewalk samples before, during, and after demolition took place in neighborhoods to measure lead content. Their findings, described in the July 2003 issue of *EHP* and the October 2005 issue of *Environmental Research*, confirmed the fears of residents: more lead was present during and after demolition than before. According to Patricia J. Tracey, the center’s community relations coordinator, the contractors hired by the city in an initiative to remove dilapidated properties have not appeared to use any defined safety measures to protect residents. “The current practices of urban demolition [in Baltimore] can be viewed as an environmental injustice to the residents who have to live with such projects and practices,” says Tracey.

These troubling findings led to the formation of the Environmental Justice Partnership in 2003, a collaboration between the center and other concerned East Baltimore community organizations, including the Maryland Institute College of Art, the Environmental Justice Partnership Community Board (comprising representatives from 10 community organizations), and staff and faculty from the Johns Hopkins Bloomberg School of Public Health.

The partnership joined with Baltimore city agencies, community organizations, residents, and public health experts in developing a new, safer demolition prototype, which includes a set of quality assurance measures to be implemented before, during, and after demolition to protect the health of residents. These measures include removing lead-containing materials from houses before they are demolished; giving residents and city agencies proper notice before engaging in building demolition; controlling dust emissions by using established wetting practices; properly containing and promptly removing debris; cleaning and repairing streets and sidewalks; and redeveloping vacant lots. The center is having ongoing meetings with community members to get feedback on the measures, and will incorporate the feedback into future development activities.

Tracey says the city is working with the community partnership to incorporate the measures into future demolition projects. East Baltimore Development, Inc., a nonprofit organization created by Baltimore’s mayor and city council to manage the revitalization of an 80-acre development of Middle East Baltimore, plans to use the safer demolition prototype in all phases of the project. Center staff have also met with Madeleine A. Shea, the new Baltimore city assistant commissioner for healthy homes, to see about getting the measures incorporated into standard city practice.

Michael A. Trush, the center’s deputy director, says the Baltimore prototype can be adapted for use in other cities, with considerable benefits. “Urban demolition is a major concern throughout low-income communities, not only in Baltimore, but in other cities in the United States,” he says. “We envision that the lessons learned and the policies developed from this project can be translated to other sites.”

## Figures and Tables

**Figure:**
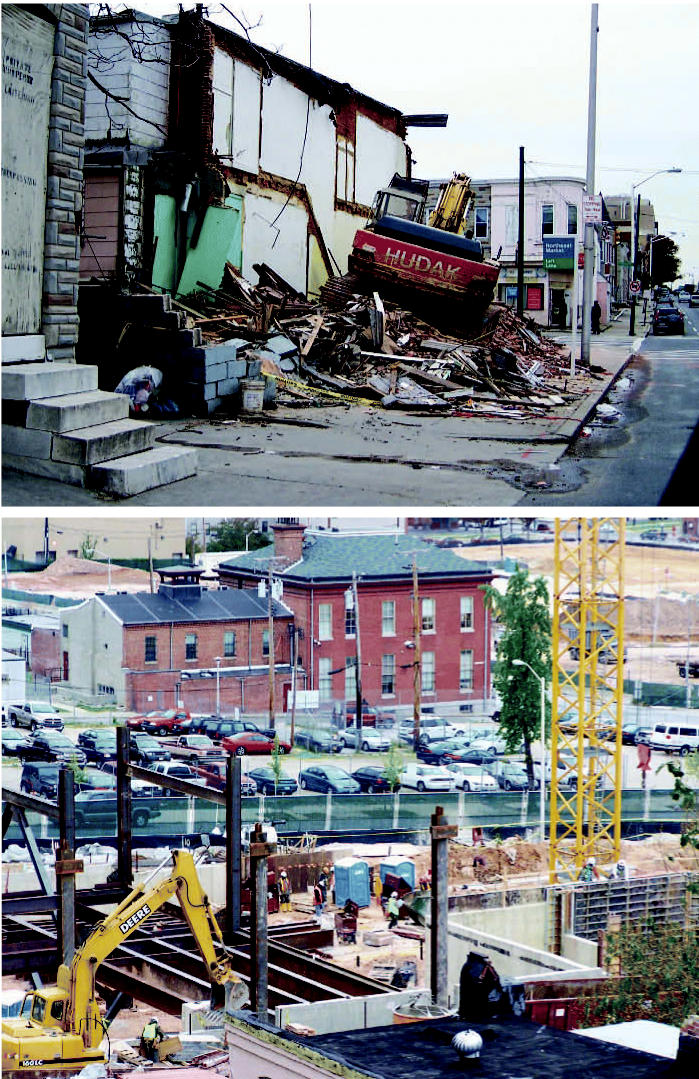
Tearing down buildings without tearing down health Concerns about environmental hazards of demolition, such as debris left on sidewalks (top), were documented by the Environmental Justice Partnership Community Board, resulting in a set of safer practices that have been used in projects such as those by East Baltimore Development, Inc. (bottom).

